# A novel role for poly(C) binding proteins in programmed ribosomal frameshifting

**DOI:** 10.1093/nar/gkw480

**Published:** 2016-06-02

**Authors:** Sawsan Napthine, Emmely E. Treffers, Susanne Bell, Ian Goodfellow, Ying Fang, Andrew E. Firth, Eric J. Snijder, Ian Brierley

**Affiliations:** 1 Department of Pathology, University of Cambridge, Cambridge, CB2 1QP, UK; 2 Department of Medical Microbiology, Leiden University Medical Center, P.O. Box 9600, 2300 RC Leiden, The Netherlands; 3 College of Veterinary Medicine, Kansas State University, Manhattan, KS 66506-5705, USA

## Abstract

Translational control through programmed ribosomal frameshifting (PRF) is exploited widely by viruses and increasingly documented in cellular genes. Frameshifting is induced by mRNA secondary structures that compromise ribosome fidelity during decoding of a heptanucleotide ‘slippery’ sequence. The nsp2 PRF signal of porcine reproductive and respiratory syndrome virus is distinctive in directing both −2 and −1 PRF and in its requirement for a *trans*-acting protein factor, the viral replicase subunit nsp1β. Here we show that the the *trans-*activation of frameshifting is carried out by a protein complex composed of nsp1β and a cellular poly(C) binding protein (PCBP). From the results of *in vitro* translation and electrophoretic mobility shift assays, we demonstrate that a PCBP/nsp1β complex binds to a C-rich sequence downstream of the slippery sequence and here mimics the activity of a structured mRNA stimulator of PRF. This is the first description of a role for a *trans*-acting cellular protein in PRF. The discovery broadens the repertoire of activities associated with poly(C) binding proteins and prototypes a new class of virus–host interactions.

## INTRODUCTION

In programmed −1 ribosomal frameshifting (−1 PRF), mRNA signals induce a proportion of translating ribosomes to slip back by 1 nucleotide (nt) into an overlapping open reading frame (ORF) and to continue translation, allowing the coordinated expression of two or more proteins from a single mRNA (1–3). First described as the mechanism by which the Gag-Pol polyprotein of the retrovirus Rous sarcoma virus (RSV) is expressed from overlapping *gag* and *pol* ORFs (4), related −1 PRF signals have been documented in many other viruses of clinical, veterinary and agricultural importance (reviewed in Ref. 5). PRF has also been increasingly recognized in conventional cellular genes of both prokaryotes and eukaryotes as well as in other replicating elements, such as insertion sequences and transposons (6,7).

Central to almost all examples of −1 PRF is the interaction of the ribosome with a stimulatory mRNA structure (a stem-loop or RNA pseudoknot) which promotes frameshifting on a stretch of homopolymeric bases known as the slippery sequence. How these RNA structures act is incompletely understood, but accumulating evidence supports the view that by presenting an unusual topology (1,2,8–11) they confound an intrinsic unwinding activity of the ribosome with consequent effects on the elongation cycle and frame maintenance (12–14). Indeed, kinetic analyses in bacterial systems indicate that stimulatory RNAs can impair movements of the ribosomal small subunit (30S) head, delaying dissociation of EF-G and the release of tRNA from the ribosome (15–17).

**Figure 1.**
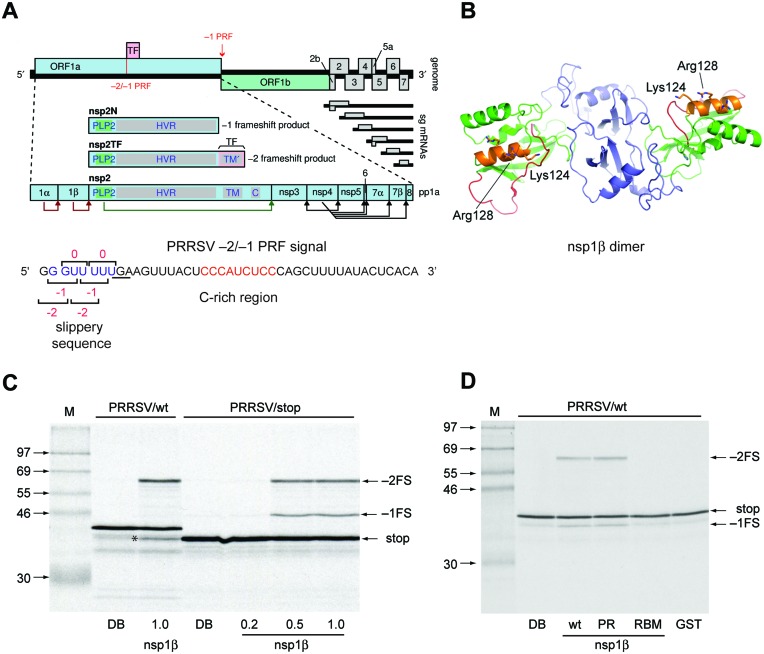
Stimulation of PRRSV −2/−1 PRF *in vitro* in RRL. (**A**) The *∼*15-kb PRRSV genome has two long 5′ ORFs, 1a and 1b encoding non-structural polyproteins (nsps) and at least eight shorter 3′ ORFs (2a-7) encoding structural proteins. ORF1a and ORF1b are translated from the genomic RNA, with translation of ORF1b depending on −1 PRF at the end of ORF1a. The TF ORF overlaps the central ORF1a region in the −2 reading frame and is accessed via −2 PRF (18). A −1 frameshift at the same site generates the nsp2N product. Shown below is the sequence of the SD01-08 RNA in the region of the −2/−1 PRF signal, with the slippery sequence (purple) and C-rich motif (red) highlighted. The −1 reading frame stop codon is underlined and codons for each of the reading frames are indicated. (**B**) Crystal structure of the PRRSV nsp1β dimer (32). The putative RNA binding domain is in orange (see text). (**C**) RNAs derived from FspI-cut pDluc PRRSV/wt, or a variant with the −1 frame stop codon (panel A) changed to UUA (PRRSV/stop), were translated in RRL in the presence of the indicated concentrations of His-tagged nsp1β (μM) or with nsp1β dilution buffer (DB). The products were resolved by 12% sodium dodecyl sulphate-polyacrylamide gel electrophoresis (SDS-PAGE) and visualized by autoradiography. ^14^C molecular size markers were also run on the gel (M). Products generated by ribosomes that do not frameshift (stop) or that enter the −1 or −2 frames are indicated. The asterisk indicates the −1 PRF product of the PRRSV/wt construct. (**D**) *In vitro* translation of pDluc PRRSV/wt mRNA in the presence of 1 μM GST-nsp1β or mutant derivatives (PR, RBM; see text). Control translations were supplemented with an equivalent concentration of expressed GST or DB alone. Translation products were analysed and quantified as above (C).

Recently, we identified a novel, highly efficient −2/−1 PRF event that functions without a recognizable stimulatory RNA secondary structure (18,19). This signal operates during translation of the genome of porcine reproductive and respiratory syndrome virus (PRRSV), a member of the family *Arteriviridae* in the order *Nidovirales* (20). The PRRSV genome (Figure 1), a positive-sense RNA molecule some 15 kb in length, harbours two PRF signals. A ‘canonical’ −1 PRF element, including a stimulatory RNA pseudoknot structure, is located at the junction of the overlapping replicase-encoding ORFs 1a and 1b and facilitates expression of an ORF1a-ORF1b fusion product. The second signal, which stimulates both −2 PRF and −1 PRF, is located within the region of ORF1a that encodes a large, multifunctional replicase subunit, non-structural protein 2 (nsp2). Here, about 20% of ribosomes translating nsp2 frameshift into the −2 reading frame to generate a transframe fusion protein (nsp2TF) comprising the N-terminal two-thirds of nsp2 and a C-terminus derived from a conserved, overlapping ORF (transframe; TF) in the −2 reading frame. A further 7% of ribosomes shift into the −1 reading frame where they immediately encounter a stop codon resulting in the synthesis of a truncated version of nsp2 termed nsp2N (19). As shown in Figure 1A, the RNA downstream of the slippery sequence (GG_GUU_UUU) used for −2/−1 PRF in PRRSV does not harbour an obvious secondary structure compatible with canonical RNA-structure-stimulated PRF. However, mutations within a conserved CCCANCUCC motif located 11 nt downstream of the shift site reduce or inhibit frameshifting, consistent with the presence of a 3′ stimulatory element of some form (18). A further novelty of the PRRSV −2/−1 PRF signal is an essential role for the 25-kDa viral replicase subunit nsp1β, which functions as a *trans*-activator of both −2 and −1 PRF (Figure 1B) (19). Nsp1β is one of the 14 subunits produced from the PRRSV replicase polyproteins. How it acts in PRF is unclear but replacement of basic residues within a highly conserved putative RNA-binding motif located in the papain-like autoproteinase (PLP) domain of nsp1β have a detrimental effect on frameshifting (19).

Through reconstitution of the PRRSV −2/−1 PRF signal *in vitro*, we show here that in addition to the viral transactivator nsp1β, efficient frameshifting requires the participation of cellular poly(C) binding proteins (PCBPs). In mammalian cells, two PCBP subsets have been described, hnRNPs K/J (21) and the αCP proteins αCP1 and αCP2, often referred to as PCBP1 and PCBP2 (22,23). The latter group also includes the more recently described paralogues PCBP3 and PCBP4 (24). PCBPs are members of the KH domain superfamily of nucleic acid binding proteins and have a wide spectrum of biological activities, including the regulation of RNA splicing, the stabilization of cellular and viral mRNAs, transcriptional activation and inhibition, and translational silencing and enhancement (reviewed in Refs. 25,26). Using *in vitro* translation and RNA binding assays, we demonstrate that a complex of nsp1β and PCBP binds to the RNA downstream of the slippery sequence and mimics the action of the more typical RNA pseudoknot stimulators of programmed frameshifting. This unprecedented frameshift-stimulatory signal provides new insights as to how the ribosomal elongation cycle can be modified by *trans-*acting protein factors. Further, it broadens the repertoire of activities associated with poly(C) binding proteins and prototypes a new class of virus–host interactions.

## MATERIALS AND METHODS

### Plasmids

Assessment of *in vitro* frameshift efficiencies employed pDluc/PRRSV wt and mutant derivatives. This vector contains the GG_GUU_UUU shift site, 5 upstream nucleotides (nt) and 66 downstream nt (79 nt in total) inserted between *Renilla* and firefly luciferase reporter genes of plasmid pDluc (27) such that −2 PRF is required for firefly luciferase expression. Protein expression vectors were as follows. PCBPs were cloned into pQE30 (Qiagen) at the BamH1/HindIII (PCBP1, 2, 3) or Sph1/HindIII (PCBP4) sites. Human PCBP1 (NM_006196.3) and PCBP2 (NM_005016.5) in pQE30 were kindly provided by Professor B. Semler, University of California, Irvine. PCBP3 (NM_021568.2 and XM_006513919.2) and PCPB4 (NM_033008.2 or XM_006713271.1) were obtained by reverse transcriptase-polymerase chain reaction (RT-PCR) using purified mRNA from mouse BV2 and human HeLa cells, repectively. Human hnRNP K/J (NM_002140.3; derived from HeLa cell mRNA by RT-PCR) was cloned into BamH1/XhoI-cut pET21a (Novagen). Nsp1β was cloned into BamH1/HindIII-cut pQE30 and BamH1/XhoI–cut pGEX6P2 (GE Healthcare). For analysis of ribosomal pausing, a 39-bp region of the PRRSV PRF region (with defective slippery sequence GGUAUUC, flanked by wild-type (WT) sequences comprising 5 nt upstream and 27 nt downstream) was cloned into the XhoI/PvuII sites of pPS0 (28). Assessment of PRF efficiencies in cultured cells employed pL-nsp1β-2, which encodes a self-cleaving nsp1β-nsp2 polyprotein under the control of a T7 promoter (19). Poliovirus P3/Leon/37 (accession number K01392.1) IRES SLIV was prepared by polymerase chain reaction (PCR) from plasmid pGemCat/PVIRES/Luc (kind gift of Dr Graham Belsham) and cloned into BamH1/EcoR1-cut pcDNA3.1 (Thermo Fisher Scientific).

### Protein expression and purification

N-terminally hexahistidine-tagged proteins expressed in pQE30 or pET21a were purified from *Escherichia coli* TG1 or BL21/DE3/pLysS cells. Single colonies were picked into Luria-Bertani broth and grown at 37°C to an OD_600_ of 0.6. Protein expression was induced by addition of isopropyl β-D-1-thiogalactopyranoside (to 0.1 mM) and continued for 2 h at 37°C (or overnight at 22°C) after which cells were pelleted and resuspended in lysis buffer (20 mM Tris, pH 7.5, 20 mM imidazole, 0.5 mM MgCl_2_, 1.4 mM 2-mercaptoethanol, 0.05% Tween 20, 500 mM NaCl, 0.1 mg/ml lysozyme). Cells were incubated on ice for 30 min and sonicated to complete lysis. Protein purification was performed using Ni-nitrilotriacetic acid (Ni-NTA; Qiagen) according to standard procedures (29). N-terminally glutathione-S-transferase (GST)-tagged nsp1β was expressed in BL21/DE3/pLysS cells as above and purified using glutathione agarose resin (ThermoFisher Scientific) according to standard procedures (30). Proteins were dialysed against 50 mM Tris, pH 7.5, 100 mM KCl, 1 mM dithiothreitol (DTT), 0.05 mM ethylenediaminetetraacetic acid (EDTA) and 5% glycerol, quantified by bicinchoninic acid assay (Pierce) and stored at −80°C until required.

### *In vitro* translation

Frameshift reporter plasmids were linearized with FspI and capped run-off transcripts generated using T7 RNA polymerase as described (31). Messenger RNAs were translated in nuclease-treated rabbit reticulocyte lysate (RRL) or wheat germ (WG) extracts (Promega) programmed with ∼50 μg/ml template mRNA. Typical reactions were of 10 μl volume and composed of 90% (v/v) RRL, 20 μM amino acids (lacking methionine) and 0.2 MBq [^35^S]-methionine. Reactions were incubated for 1 h at 30°C and stopped by the addition of an equal volume of 10mM EDTA, 100 μg/ml RNase A followed by incubation at room temperature for 20 min. Samples were prepared for sodium dodecyl sulphate-polyacrylamide gel electrophoresis (SDS-PAGE) by the addition of 10 volumes of 2× Laemmli's sample buffer, boiled for 4 min and resolved by SDS-PAGE. Dried gels were exposed to a Cyclone Plus Storage Phosphor Screen (PerkinElmer), the screen scanned using a Typhoon TRIO Variable Mode Imager (GE Healthcare) in storage phosphor autoradiography mode and bands were quantified using ImageQuant™TL software (GE Healthcare). The calculations of frameshifting efficiency (%FS) took into account the differential methionine content of the various products and %FS was calculated as % −1FS = 100 × [IFS1/MetFS1) / [IS/MetS + IFS1/MetFS1) + IFS2/MetFS2)] and similarly for % −2FS. In the formula, the number of methionines in the stop, −1FS and −2FS products are denoted by MetS, MetFS1 and MetFS2 respectively; while the densitometry values for the same products are denoted by IS, IFS1 and IFS2, respectively. All frameshift assays were carried out a minimum of three times and the measured frameshift efficiencies (±SEM) are provided in the Supplementary Data.

### Ribosome pausing assays

WG *in vitro* translation reactions (30 μl) were supplemented with 1 μM nsp1β, PCBP2 (or KH mutant derivative) or both proteins and programmed with mRNAs derived from AvaII-cut pPS0/PRRSV WT or pPS0/PRRSV CC2. Reactions were incubated at 18°C for 5 min prior to addition of edeine to 5 μM. Aliquots (1.5 μl) were subsequently withdrawn at intervals post-edeine addition, mixed with an equal volume of 10mM EDTA, 100 μg/ml RNase A and placed on ice. At the end of the time-course, samples were analysed by SDS-PAGE.

### Electrophoretic mobility shift assay (EMSA)

Short, ^32^P-labelled template RNAs (58 nt) containing the PRRSV PRF region (with slippery sequence precisely at the 5′ end and C-rich region towards the centre) were prepared by T7 transcription of a PCR product generated using primers flanking the frameshift region, with the 5′ primer containing the T7 polymerase promoter sequence. Test proteins were diluted where necessary in dilution buffer (DB) (5 mM Tris pH 7.5, 100 mM KCl, 1 mM DTT, 0.05 mM EDTA, 5% glycerol) and added to reactions (10 μl final volume) alongside electrophoretic mobility shift assay (EMSA) buffer (10 mM Hepes pH7.6, 150 mM KCl, 2 mM MgCl_2_, 1 mM DTT, 0.5 mM adenosine triphosphate, 5% glycerol, 100 μg/ml porcine tRNA, 10U RNase inhibitor per ml), after which the radiolabelled probe was introduced. After incubation at 30°C for 10 min, samples were loaded promptly onto 4% acrylamide non-denaturing gels (acrylamide:bisacrylamide ratio 10:1) and run at 175V at room temperature until free and bound RNA species were resolved. Gels were fixed for 15 min in 10% acetic acid, 10% methanol, dried and exposed to X-ray film.

### Pull down assays

For each protein tested, 40 μl of a 50% suspension of glutathione-agarose beads was centrifuged (500 *g*, 5 min), the supernatant removed and the beads washed sequentially with 200 μl water and thrice with GST wash buffer (50 mM Tris pH 7.6, 100 mM KCl, 5 mM MgCl_2_, 1 mM DTT). Following incubation with nsp1β (6 μg) for 1 h at 4°C on a rotating wheel, the protein was removed and the beads washed three times with GST wash buffer. Test protein (2 μg) was added to the beads and the incubation and washing steps repeated, with the GST wash buffer supplemented with 0.1% NP-40. After removal of the final wash, 25 μl 4 × Laemmli's sample buffer was added, the beads boiled for 5 min and supernatants analysed by SDS-PAGE.

### siRNA-mediated knockdown

Details are provided in Supplementary Data.

## RESULTS

### Reconstitution of the PRRSV −2/−1 PRF signal *in vitro*

*Trans-*activation of PRRSV −2/−1 PRF by nsp1β was previously demonstrated by co-expression of nsp1β and nsp2 in cultured cells and by site-directed mutagenesis of the viral genome (18,19). To study the phenomenon *in vitro*, a 79-bp fragment encompassing the slippery sequence and C-rich region was subcloned between the *Renilla* and *firefly* luciferase genes of a frameshift-reporter plasmid (pDluc) such that expression of the downstream cistron (*fluc*) was dependent upon −2 PRF within the inserted PRRSV sequences. In RRL *in vitro* translations, mRNAs transcribed from pDluc/PRRSV specified the synthesis of only the product of the 5′ cistron of the reporter mRNA (*Rluc*) and no frameshifting was evident (Figure 1C). However, supplementation with recombinant, purified His-tagged nsp1β yielded two additional proteins, the most abundant corresponding to the product of −2 PRF. The second protein migrated more rapidly than that of *Rluc* and we surmised that this protein corresponded to the product of a −1 PRF, as a −1 frame stop codon is present immediately downstream of the slippery sequence (underlined in Figure 1A). To confirm this, the −1 frame stop codon (UGA) was changed to UUA, which extends the −1 reading frame by 56 codons and simultaneously introduces a 0-frame stop codon UAA thus shortening the *Rluc* gene. As expected, this point mutation increased the size of the predicted −1 PRF product, and slightly reduced the size of the Rluc protein (stop). These experiments indicate that the 79-nt region cloned into pDluc likely contains all of the *cis*-acting elements required for PRRSV −2/−1 frameshifting and that supplementation of the RRL translations with nsp1β alone is sufficient to *trans*-activate frameshifting to levels similar to those seen in infected cells (−2 PRF, 17%; −1 PRF, 7%; see Supplementary Table). The stimulation of PRF by nsp1β was specific to the PRRSV signal in that the protein had no effect on frameshifting at a variety of well-established sites of viral −1 PRF (Supplementary Figure S1). Furthermore, the protein was unable to promote stop codon readthrough at variants of the PRRSV signal with an in-frame stop codon placed within the slippery sequence at the position that would be decoded in the peptidyl- (P) or aminoacyl- (A) site of the ribosome (Supplementary Figure S1).

The crystal structure of nsp1β (Figure 1B) reveals an overall ellipsoidal structure consisting of six α-helices and seven β-strands with two major domains, a 48-amino-acid N-terminal domain (NTD) and a C-terminal papain-like cysteine protease (PLP1β) domain (32). Within the latter domain, we previously identified a conserved motif _123_GKYLQRRLQ_131_ (19) as a potential RNA binding motif (RBM) that forms one of three α helices in the region between the catalytic residues of PLP1β (C_96_ and H_165_; Ref. 19). To investigate the importance of these activities to *trans*-activation of PRF, amino acid substitutions were introduced into the protease active site (C96S; PR) or within the putative nsp1β RBM (K_124_A/R_128_A, mutant RBM; previously described as mutant 1βKO (Li *et al*., (19)) and the expressed variants tested in RRL (Figure 1D). In this experiment, the protease-defective variant retained full activity, ruling out the involvement of the protease activity of nsp1β in the stimulation of PRF. In contrast, the RBM mutation prevented frameshifting, supporting a role for RNA binding by nsp1β.

### A requirement for Poly(C) binding proteins in PRRSV −2/−1 PRF

**Figure 2.**
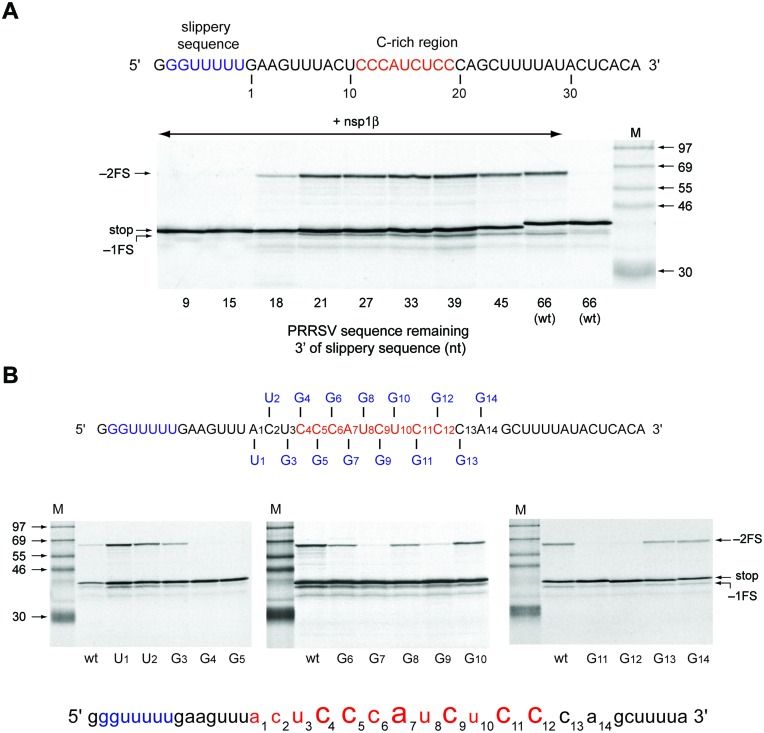
The C-rich region is essential for PRF. (**A**) A series of 3′ deletions were engineered into pDluc PRRSV/wt and transcribed mRNAs translated in RRL in the presence of added nsp1β (1 μM). Products were analysed by SDS-PAGE as detailed in the legend to Figure 1. The number below each lane represents the length of the PRRSV-specific RNA sequence remaining downstream of the slippery sequence, of which 21 nt were sufficient for efficient −2/−1 PRF in this assay. (**B**) Individual point mutations were introduced in and around the C-rich region (red) within pDluc PRRSV/wt and transcribed mRNAs translated and analysed as above. The effect of each mutation on PRF is illustrated on the sequence below the gel, with a larger font size reflecting a greater inhibition of PRF.

To assess the role in PRF of sequences 3′ of the slippery sequence, a series of in-frame deletions were introduced into pDluc/PRRSV, starting from the 3′ end of the inserted PRRSV sequence. RNAs transcribed from these plasmids were translated in RRL in the presence of nsp1β (Figure 2A). PRF was found to be unaffected until the length of the PRRSV sequence downstream of the slippery sequence fell below 21 nt, at which point PRF was reduced (18 nt) or abolished (15 or 9 nt). In the latter constructs, the C-rich region is compromised, consistent with an important role in PRF. Similar results were previously observed in transfected cultured cells (Li *et al*., (19)). To investigate the function of the C-rich region specifically, we prepared a library of individual point mutations within, and flanking, the C-rich region and assessed their effect on PRF (Figure 2B). It was found that changing any of the C residues to G had a detrimental effect on PRF, with the exception of the final residue (C_13_, 3′ of the conserved sequence), where the effect was more modest. As an explanation for these observations, we speculated that the RNA downstream of the slippery sequence may interact with PCBPs. The ‘minimal’ region of PRRSV RNA required for full activity in PRF (21 nt) has only two C-rich patches, whereas previously established PCBP-binding consensus sequences more commonly have three (or more) C-triplets, potentially with each triplet binding one KH domain ((33); see below). However, PCBP1 and PCBP2 are amongst several candidates reported to associate with GST-tagged nsp1β in association screens, and a direct physical interaction between the two has been shown (34,35). PCBPs could therefore bind to the PRRSV mRNA by virtue of an association with nsp1β.

**Figure 3.**
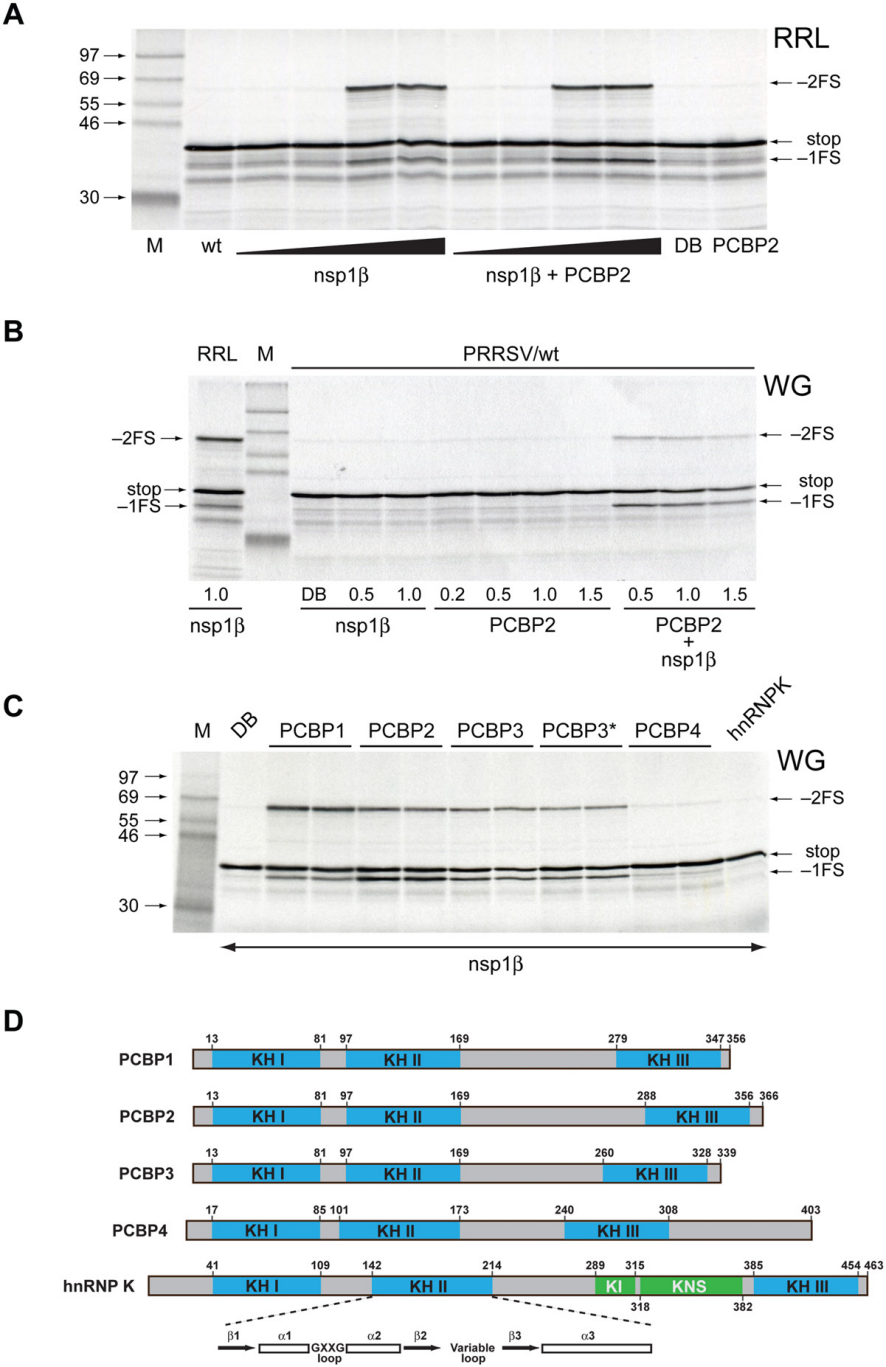
A requirement for poly(C) binding proteins in PRRSV −2/−1 PRF. (**A**) *In vitro* translation of pDluc PRRSV/wt mRNA in RRL alone (wt), or in the presence of PCBP2 alone (0.7 μM), nsp1β (from 0.1 to 1 μM) or both proteins (PCBP2, 0.7 μM; nsp1β from 0.1 to 1 μM). A control translation was supplemented with PCBP2 dilution buffer (DB). (**B**) *In vitro* translation of pDluc PRRSV/wt mRNA in WG extract in the presence of the indicated concentrations of nsp1β (μM), PCBP2 (μM) or both proteins (PCBP2, from 0.5 to 1.5 μM; nsp1β, 1 μM). A control translation of pDluc PRRSV/wt mRNA in RRL is also shown. (**C**) *In vitro* translation in WG extract of pDluc PRRSV/wt mRNA in the presence of nsp1β (1 μM) with added PCBP1, 2, 3 or 4 (at 0.5 and 1 μM), hnRNPK (1 μM) or DB. Note PCBP3s were of mouse origin, with PCBP3 corresponding to accession number NM_021568.2 and PCBP3* to XM_006513919.2. (**D**) Multidomain structure of the PCBP family showing location of KH domains (numbered with respect to human sequence) and secondary structure of individual KH domains. Adapted from Refs. (25 and 26).

To explore the potential role of PCBPs in PRRSV PRF, we translated the pDluc/PRRSV mRNA in RRL reactions supplemented with nsp1β, His-tagged human PCBP2, or both proteins (Figure 3A). We found that PCBP2 alone did not affect PRF (right hand lane), but when added together with nsp1β, synthesis of the −1 PRF product was stimulated some 3-fold (see Supplementary Table). We reasoned that if the ‘active’ *trans-*acting stimulator of PRF in PRRSV is a complex of nsp1β and PCBP, the RRL system may already contain an abundant form of PCBP, but one which would, in complex with nsp1β, preferentially induce −2 PRF. In this experiment, supplementation with PCBP2 may have generated nsp1β–PCBP2 complexes that could preferentially promote −1 PRF. We therefore translated the PRRSV frameshift reporter mRNA in the WG *in vitro* translation system on the basis that this lysate might contain fewer endogenous PCBPs, or PCBPs of sufficient evolutionary divergence to preclude any interactions with nsp1β. Consistent with our hypothesis, PRF at the PRRSV signal in WG was found to be completely dependent upon the addition of both nsp1β and PCBP2, with neither protein alone having any frameshift-stimulatory activity in this system (Figure 3B). Further, supplementation of WG translations with PCBP2 led preferentially to a −1 PRF, whereas with human PCPB1, −2 PRF was most evident (Figure 3C), suggesting that the abundant form in RRL is PCBP1. We also tested mouse PCBP3, human PCBP4 and mouse hnRNP K/J in WG translations (Figure 3C and D). Of these three proteins, only PCBP3 was able to promote frameshifting, with both −2 and −1 PRF products observed at similar levels (Figure 3C). We confirmed the involvement of PCBP1 and 2 in frameshifting in cultured cells using siRNAs (Supplementary Figure S2). Knockdown of PCBPs individually or together substantially reduced PRF, with PCBP1 noticeably affecting −2 PRF, and PCBP2, −1 PRF, consistent with the results of the WG assays above. No effect on PRF was seen with siRNAs targeting PCBP3, PCBP4 or hnRNP K/J, although we could not verify knockdown of PCBP3 due to lack of a reactive antibody.

### Synergistic binding of nsp1β and PCBP to the PRRSV −2/−1 PRF signal

**Figure 4.**
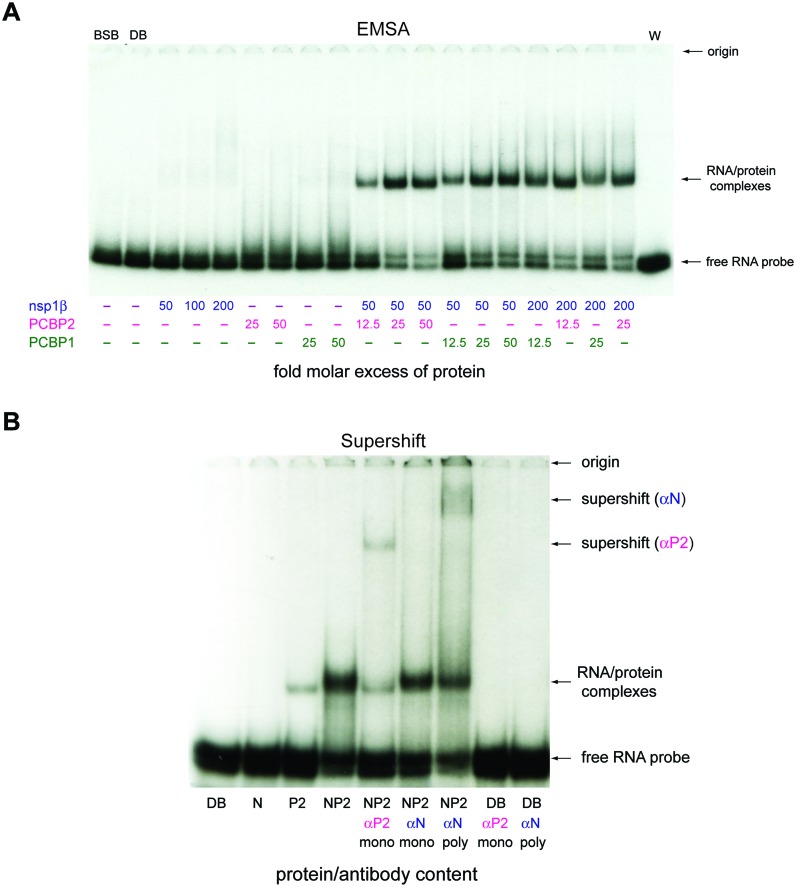
Synergistic binding of nsp1β/PCBP to the PRRSV mRNA. (**A**) EMSA analysis of binding of nsp1β, PCBP1 or PCBP2 (alone or in combination) to a short (58 nt) ^32^P-labelled *in vitro* transcript containing the PRRSV PRF signal. After incubation at 30°C for 10 min, reactions were loaded onto a 4% non-denaturing polyacrylamide gel and, following electrophoresis, the gel was fixed, dried and subjected to autoradiography. Numbers below lanes show fold molar excess of protein(s) with respect to the radiolabelled RNA (10 nM). In lanes BSB, DB and W, RNA was incubated alone with band-shift buffer, protein dilution buffer or water, respectively (see ‘Materials and Methods’ section). (**B**) The composition of RNA–protein complexes was investigated by supershift assay. Complexes were prepared with 10 nM RNA and a 50-fold molar excess of nsp1β (N) and PCBP2 (P2) and subjected to EMSA directly (N, P2, NP2), or after incubation with monoclonal (mono) or polyclonal (poly) antisera raised against PCBP2 (αP2, mono) or nsp1β (αN, mono or poly). As controls, the radiolabelled RNA probe was incubated with either DB, the αP2 mono or the αN poly.

RNA binding by the PRRSV PRF *trans*-activators was investigated using EMSAs. A short (58 nt) ^32^P-labelled transcript encompassing the minimal PRRSV frameshift-stimulatory region was incubated with nsp1β, PCBP1 (or PCBP2) or both proteins, prior to electrophoresis on a 4% non-denaturing polyacrylamide gel. As shown in Figure 4A, a single RNA–protein complex was observed, the formation of which required both nsp1β and PCBP1 (or PCBP2). Using a broader range of PCBP2 concentrations at saturating levels of nsp1β, the estimated *K*_d_ of the interaction was found to be 130 nM (Supplementary Figure S3). In the presence of PCBP2, the PR mutant of nsp1β was able to bind the target RNA indistinguishably from the WT protein, but the RBM mutant gave a smeary pattern, indicative of weak, non-specific, RNA binding (Supplementary Figure S3). As a control, a version of the RNA probe mutated in the C-rich region (CC2, Ref. 18) was also tested, and no nsp1β/PCBP2 binding was evident (Supplementary Figure S3). A supershift assay was carried out to confirm nsp1β and PCBP association with the RNA in the complex. The PRRSV RNA was incubated with nsp1β and PCBP2 and subsequently with specific antibodies targeting either protein prior to EMSA. As shown in Figure 4B, in lanes from reactions with an added anti-PCBP2 monoclonal antibody, an additional band of slower mobility was seen consistent with the presence of PCBP2 in the RNA/protein complex. A monoclonal antibody targeting the NTD of nsp1β did not yield a novel EMSA product, but a more slowly migrating species was observable with a polyclonal anti-nsp1β serum. These observations support the view that the RNA/protein complex seen contains both proteins.

We carried out competition experiments to assess whether the WT PRRSV mRNA, the PRF-defective CC2 variant or a known PCBP2-binding RNA from poliovirus (internal ribosome entry signal [IRES] stem-loop IV; Ref. 36) could compete with the frameshift reporter mRNA for PCBP2 binding in *in vitro* translation assays in RRL supplemented with nsp1β (Supplementary Figure S4). Consistent with expectation, a dose-dependent reduction in frameshifting efficiency was observed upon addition of short, uncapped transcripts containing the WT PRRSV PRF signal or IRES stem-loop IV RNAs, but not with the control mRNA with the C-rich stretch mutated.

### Differential contribution of PCBP KH domains in PRF

**Figure 5.**
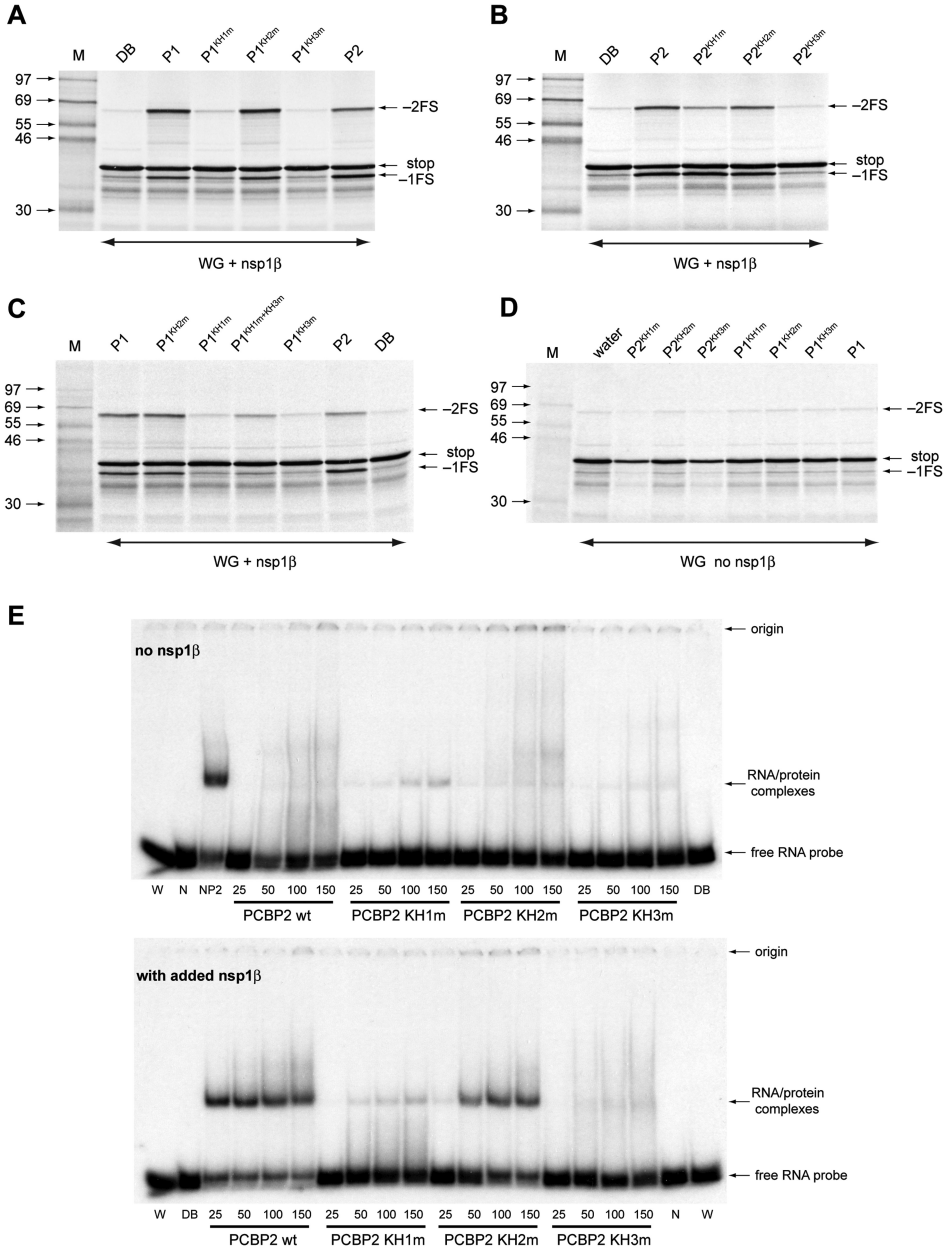
Role of PCBP1/2 KH domains in PRRSV PRF. (**A**) *In vitro* translation in WG extract of pDluc PRRSV/wt mRNA in the presence of nsp1β (1 μM) and PCBP1, PCBP2 or PCBP1 KH domain mutants (P1 KH1-3m; 1 μM). DB is dilution buffer. (**B**) Analysis of PCBP2 KH domain mutants, details as in panel (A). (**C**) Compensatory effect on −2/−1 PRF of supplementing a WG translation with both PCBP1 KH1m and PCBP1 KH3m, details as in panel (A). (**D**) Individual PCBP1/2 KH domain mutants do not stimulate PRF in the absence of nsp1β, details as in panel (A). (**E**) EMSA analysis of PCBP2 KH domain mutants. Binding of the KH domain mutants to the PRRSV RNA (10 nM; details in Figure 4) was investigated in the absence or presence of added nsp1β (1 μM).

PCBP family members have three KH domains, an adjacent pair (KH1 and KH2) close to the N-terminus and a third (KH3) towards the C-terminus, separated from KH1 and KH2 by an intervening sequence of variable length (Figure 3D). To test the involvement of individual KH domains in PRF, amino acid substitutions (to GDDG) were introduced within the conserved, hallmark GxxG loop in each domain, which forms part of the RNA binding domain. These mutations have been shown previously to abolish RNA binding activity of the individual domain without compromising its stability (37). As shown in Figure 5A and B, mutation of the KH2 domain of PCBP1 or PCBP2 had little, if any, effect on frameshifting, whereas the KH3 domain mutation essentially abolished *trans*-activation. The KH1 domain mutation had a more varied phenotype. With PCBP1, frameshift-stimulation was greatly reduced, but for PCBP2, −2 PRF was diminished four-fold, but full activity in −1 PRF was retained. Further, whilst addition of PCBP1^KH1m^ or PCBP1^KH3m^ alone was unable to stimulate PRF, when added together, a partial restoration of both −2 and −1 PRF (to about 20% of the WT value) was observed (Figure 5C). Note that in all assays, frameshift-stimulation remained dependent upon addition of nsp1β (Figure 5D).

From the lack of effect of the KH2 mutation on PRF, we conclude that interaction of this domain with RNA does not occur (or is unnecessary for PRF) and that mutations within the GxxG loop of KH2 do not compromise the specific interaction with nsp1β. However, the KH1 and KH3 mutations likely modify the interaction of the protein with RNA, and conceivably, with nsp1β. EMSA analysis confirmed the predicted effects on RNA binding, which was reduced in the case of PCBP2^KH1m^, abolished with PCBP2^KH3m^ and largely unaffected for PCBP2^KH2^ (Figure 5E). Similar results were seen with PCBP1 (Supplementary Figure S5). To assess the capacity of the various PCBPs to bind nsp1β independently of RNA, a pull-down assay was utilized (Supplementary Figure S6). Here, immobilized GST-nsp1β was incubated with individual His-tagged PCBPs and, after a detergent wash, bound protein was eluted for analysis by immunoblotting. In comparison to WT PCBP2, the binding of PCBP2 bearing any of the three KH domain mutations to GST-tagged nsp1β was unaffected, indicating that the effects on PRF are related to changes in RNA binding rather than a reduced capacity to associate with nsp1β. Indeed, PCBPs 1–4 all bound nsp1β in this assay, thus the inability of PCBP4 to *trans*-activate PRF at the PRRSV signal has some other explanation. Importantly, GST-tagged nsp1β RBM was able to pull-down PCBP2, indicating that the defect in this mutant is not in the capacity to associate with its protein partner.

### Mechanistic insights into protein-stimulated ribosomal frameshifting

#### Spacing

**Figure 6.**
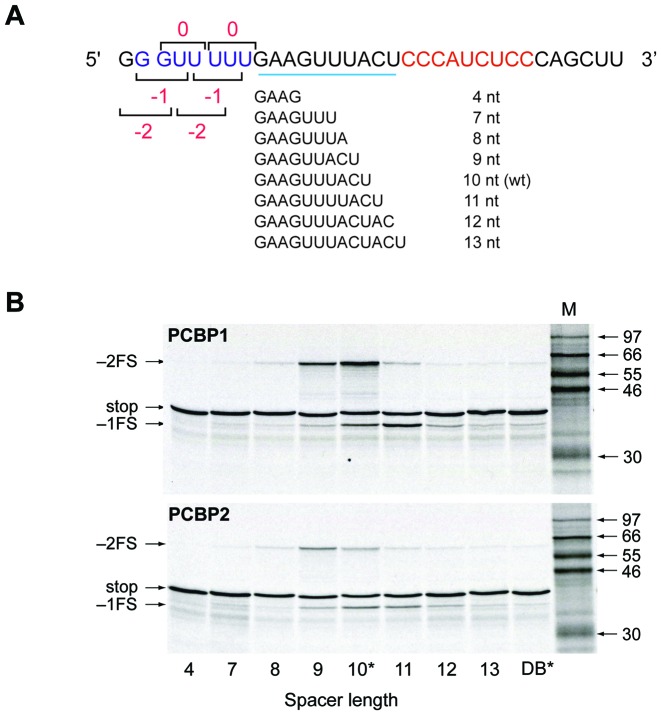
Spacer length is critical to PRRSV PRF magnitude and direction. (**A**) The spacer sequence separating slippery sequence and C-rich region of pDluc PRRSV/wt mRNA (underlined in blue) was varied from 4 to 13 nt (from WT [wt] of 10 nt, asterisked) as indicated. Where necessary, the Fluc reading frame reporting −2 PRF was maintained by inserting one or two additional nucleotides immediately downstream of the inserted PRRSV sequences in pDluc. (**B**) RNAs were translated in WG in the presence of nsp1β (1 μM) and PCBP1 or PCBP2 (1 μM). Products were analysed as in Figure 1.

A hallmark of RNA structure-dependent PRF signals is the necessity to maintain a precise spacing between slippery sequence and stimulatory RNA to position the ribosome appropriately at the slip-site (38,39). The importance of spacing in PRRSV frameshifting was assessed in WG translations programmed with reporter mRNAs with spacer lengths of 4–13 nt (Figure 6). Efficient PRF was seen over a narrow range (9–11 nt), with shorter spacers leading preferentially to −2 PRF and longer spacers preferentially to −1 PRF. This pattern of frameshifting is highly reminiscent of that observed with stimulatory RNAs and suggests that the nsp1β/PCBP complex functionally mimics such RNAs. The optimal spacer length was subtly different for the two PCBPs, with maximal PRF (−2 and −1) seen at longer spacer lengths for added PCBP1 compared with PCBP2. Thus the capacity of the various PCPBs to promote frameshifting into a particular reading frame is likely set by the position and/or conformation of the protein complex on the template, affected by subtle differences in the position of KH domains within the proteins which influences ‘effective’ spacer length.

#### Pausing

**Figure 7.**
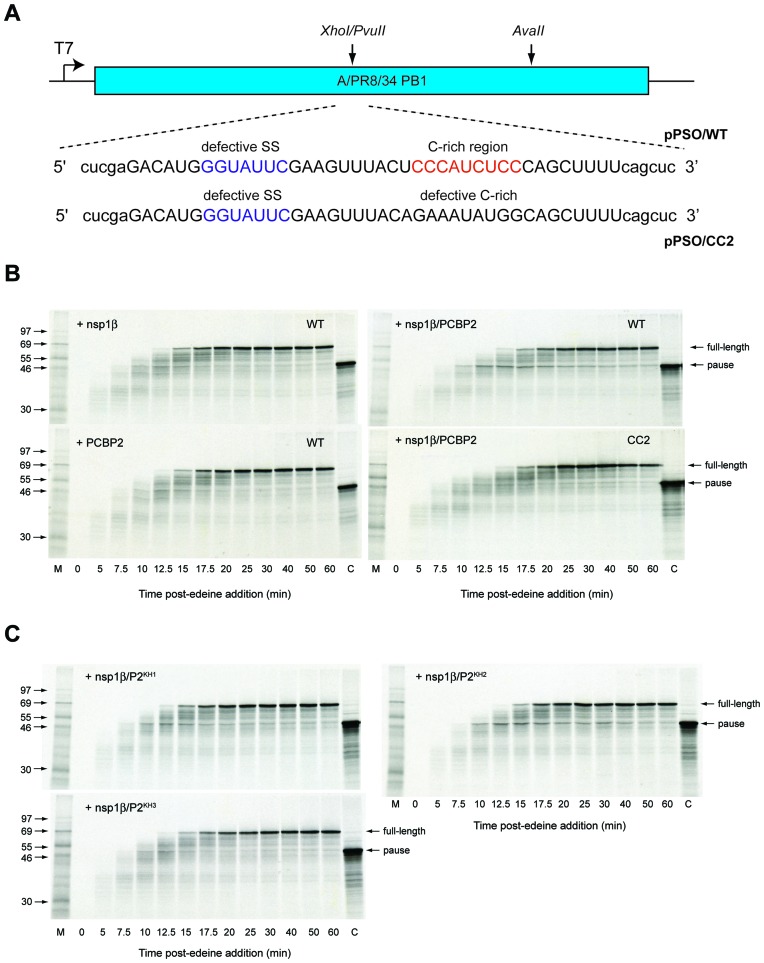
Ribosomal pausing at the PRRRSV −2/−1 PRF signal. (**A**) The PRRSV WT (WT) C-rich region with a mutated slippery sequence (as indicated) was cloned into plasmid pPS0 (28) to generate plasmid pPS0/WT. A control plasmid (pPS0/CC2) additionally contained mutations in the C-rich region (see text). (**B**) RNAs derived from AvaII-cut pPS0/WT or pPS0/CC2 were translated in WG extract at 18°C for 5 min prior to addition of edeine to 5 μM. Aliquots were removed at various times post-edeine addition, translation stopped and products resolved by 12% SDS-PAGE. The expected size of the ribosomal pause product (pause) was marked by translating an mRNA produced from XhoI-cleaved pPS0. Translations were supplemented with 1 μM nsp1β, PCBP2 or both proteins as indicated. (**C**) Ribosomal pausing assays were carried out as described in panel (B) above. Here, AvaII-cut pPS0/WT mRNA was translated in WG extract supplemented with 1 μM nsp1β and the KH domain mutants of PCBP2, as indicated.

Ribosome pausing at structured RNA stimulators of PRF has been widely documented and may play a mechanistic role (2). We tested whether the nsp1β/PCBP2 complex could also induce pausing by cloning the PRRSV signal (with a defective slippery sequence) in-frame within a reporter gene and translating *in vitro* derived mRNAs in the WG system. The extent of pausing was estimated by comparing the levels of a translational intermediate corresponding to pausing at a bound nsp1β/PCBP2 complex with that of the full-length polypeptide produced during a time course in which translation was synchronized by the addition of edeine, a potent inhibitor of initiation, 5 min after the start of the reaction. A control mRNA was prepared from plasmid template linearized at the position of the inserted PRRSV signal to serve as a pause marker. As can be seen in Figure 7, pausing was clearly evident in the presence of nsp1β/PCBP2, but far less so with either protein alone, or on an mRNA lacking the C-rich region. The appearance of the pause product was transient (albeit visible over several minutes), consistent with its identity as a genuine intermediate rather than a dead-end product. In Figure 7C, further pausing assays were carried out to examine the activity of the PCBP2 KH domain mutants (in the presence of nsp1β). We found a positive correlation between the extent of pausing observed and the activity of the three mutants in specific RNA binding, namely a little for PCBP2^KH1m^, close to WT levels for PCBP2^KH2m^ and much less pausing for PCBP2^KH3m^.

## DISCUSSION

A role for PRRSV nsp1β protein in PRF was the first description of a protein *trans-*activator of this process (19). Here, we show that the active frameshift-stimulatory element in fact comprises a complex of viral nsp1β and a cellular poly(C) binding protein. PCBPs have been shown to regulate gene expression at multiple levels, including transcription, mRNA processing, mRNA stabilization and translation (reviewed in Refs. 25,26). Hitherto, a role for PCBPs in protein synthesis has been limited to translation initiation, mostly in the recruitment of ribosomes to viral IRESes (see Ref. 40). Their activity in stimulating PRF in PRRSV is the first example of a role for PCBPs in modulating translational elongation.

The induction of PRF by the nsp1β–PCBP complex has clear parallels with *cis-*acting structured RNA stimulators, including the capacity to induce a ribosomal pause and the necessity for maintaining a precise distance between slippery sequence and stimulatory element. In RNA structure-dependent PRF, interaction of the stimulatory RNA with the ribosome impairs movement of the small subunit head during translocation, destabilizing the hybrid state and elevating energy barriers corresponding to subsequent substeps of translocation (15–17). Completion of translocation is accelerated by slippage of tRNAs into the −1 frame, thus promoting PRF. At the PRRSV signal, the bound nsp1β–PCBP complex would also be in close proximity to a ribosome actively decoding the slippery sequence, facilitating interactions between them. In this light, it may be significant that in pull down assays, nsp1β has been shown to interact with rpS14 (34), a protein immediately adjacent to a component (rpS3) of the putative ribosome-associated mRNA unwinding activity (13) and PCBP1 is known to interact with RACK1 (41), a ribosome-associated protein located close to the mRNA entry channel. Details of the molecular interactions that take place upon encounter of elongating ribosomes remain to be determined.

The geometry and stoichiometry of the nsp1β–PCBP complex on the PRRSV mRNA is uncertain and difficult to predict. Nsp1β exists as a dimer in solution (32), PCBPs have been shown to be capable of homo- and hetero-multimerization and RNA binding by PCBPs could reflect interaction with single or multiple KH domains (42–44). Binding assays with optimized RNA targets generated by *in vitro* SELEX have revealed that a tandem array of three C-patches maximizes PCBP2 binding to its RNA target (45) and several established PCBP binding sites contain three or more C-patches (33,46). However, the sequence motifs implicated in PCBP binding from gene expression analysis of human cells (47,48) and *Caenorhabditis elegans* (49) comprise predominantly two adjacent C-rich patches, although it is not known how strongly PCBPs bind to these targets, nor whether any other cellular proteins participate in the interactions. The EMSA data presented here reveal that alone, PCBP1 and PCBP2 bind weakly to the PRRSV C-rich region (which has two C-patches), requiring nsp1β for stable binding. Similarly, nsp1β binds RNA very weakly, but is evident in complexes (with PCBPs) from supershift assays. Stable complex formation on the mRNA thus likely depends upon conformational changes in one or both molecular partners and potentially, direct contact between nsp1β and mRNA. In support of the latter, we found that amino acid substitutions in the putative RBM) of nsp1β greatly weakened the association of the complex with RNA, without apparent effect on the interaction with PCBP2. Yeast two-hybrid studies (35) have shown that the reciprocal interaction of nsp1β and PCBP2 requires the C-terminus of nsp1β, including the protease domain (PLP1β) and C-terminal extension, and the KH2 domain of PCBP2. In light of this, we hypothesize that the association of nsp1β with KH2 would not preclude binding of nsp1β to RNA. An alternative interpretation is that nsp1β RBM fails to induce a conformational change in PCBPs necessary for stable RNA association.

Our model for PRRSV −2/−1 PRF is that PCBPs interact with the PRRSV mRNA through KH1 and KH3, with each domain contacting one of the two C-patches (CCCA and CUCC) within the C-rich region. The KH2 domain itself is not associated with the RNA, serving rather to bind nsp1β, with the complex poised to subvert the elongating ribosome. In its capacity to stall ribosomes, the nsp1β–PCBP complex joins a growing list proteins that have been shown to modulate the elongation step of protein synthesis. These include Stm1 of *Saccharomyces cerevisiae*, which inhibits translation after 80S formation (50), the fragile X mental retardation protein, which reversibly stalls ribosomes on its target mRNAs (51) by binding directly to the ribosome (52), the HIF-1α mRNA-associated cytoplasmic polyadenylation element binding protein 2, which binds eEF2 and slows elongation (53) and the PUF-Ago-eEF1A complex that attenuates elongation after the nascent peptide emerges from the ribosomal exit tunnel (54). Stalling of ribosomes by the nsp1β–PCBP complex, perhaps as a consequence of a direct interaction with the unwinding centre of the small subunit, could promote PRF in a manner similar to that described for structured RNA stimulators.

Not all PCBPs were capable of stimulating PRF. The lack of activity of PCBP4 and hnRNP K/J is potentially a consequence of differences in overall size and domain organization. PCBP1, 2 and 3 are globally similar, with KH domains located at analogous positions in the protein, whereas the linker separating KH2 and KH3 is shorter in PCBP4 and much longer in hnRNP K/J (Figure 3D). These differences did not affect association of PCPB4 with nsp1β, but could affect the strength of binding of individual KH domains to RNA or alternatively, preclude generation of a correctly oriented or positioned complex on the mRNA. With the KH domain mutants of PCBP1 and PCBP2, we largely observed an excellent correlation between activity in RNA binding, ribosomal pausing and the capacity to induce PRF. However, we saw differential activity in PRF of the KH1 domain mutants of PCBP1 and PCBP2, with the former barely active yet the latter retaining function in promoting −1 PRF, but with reduced −2 PRF. When RNA association was examined by EMSA, PCBP1^KH1m^ and PCBP2^KH1m^ bound to the PRRSV RNA with similar affinity and in an nsp1β-dependent manner, although binding was noticeably reduced in comparison to the WT proteins. This suggests that PCBP1^KH1m^ and PCBP2^KH1m^ still associate with the RNA, but the stability, conformation or position of the complex may be changed such that only in the case of PCBP2^KH1m^ is PRF possible, and only −1 PRF (which is the predominant frameshift event for WT PCBP2 at the natural spacing distance). As noted in the ‘Results’ section, compensatory activity in PRF was detected upon mixing the individually inactive PCBP1^KH1m^ and PCBP1^KH3m^, suggesting that two (or more) PCBPs can cooperate to stimulate frameshifting. The differential activity of PCBP1^KH1m^ and PCBP2^KH1m^ could reflect an altered capacity to homo-oligomerize.

The *trans*-activation of −2/−1 PRF by nsp1β/PCBP has intriguing parallels with a recently described translational readthrough event in human vascular endothelial growth factor A (VEGFA) mRNA (55). Here, low-frequency stop codon readthrough at the end of the pro-angiogenic VEGFA coding sequence appends an additional 22 amino acids to the C-terminus, generating the VEGF-Ax isoform which possesses anti-angiogenic activity. The readthrough event is promoted by the binding of hnRNP A2/B1 to a recognition element 10 nt downstream of the recoded VEGFA stop codon. Similar to PCBPs, hnRNP A2/B1 is known to associate with nascent cellular RNAs and influence their localization, maturation and function (56). How it acts to stimulate readthrough is unknown, but it is striking that both hnRNP A2/B1 and the nsp1β/PCBP complex promote their respective recoding events from a similar distance 3′ of the ribosome, suggesting that they may contact the ribosome directly. How they subvert translation likely differs in each case as we were unable to uncover any stop codon readthrough activity of the nsp1β/PCBP2 complex (Supplementary Figure S1).

Our description of a role for PCBPs in ribosomal frameshifting adds to a growing number of examples of viral subversion of PCBP function. In the best characterized system, poliovirus, the capacity of PCBPs to associate at two discrete genomic RNA locations and in concert with different protein partners is critical to almost all aspects of the virus replication cycle (reviewed in Ref. 57). Whether such complex regulatory circuits operate during PRRSV replication and transcription remains to be determined. PCBP2 has been shown to associate with the 5′ untranslated region of the PRRSV genome and may be involved in genome replication or translation initiation (34,35). Furthermore, the relative abundance and location of PCBPs in the cytoplasm of infected cells could be important in determining the precise ratio of non-frameshifted (full-length nsp2) and *trans-*frame (nsp2TF, nsp2N) products at various stages of the replication cycle.

Presently, stop codon readthrough in VEGFA (55) and PRF in PRRSV (19, this work) represent the only two examples of specific modulation of translational decoding (recoding) mediated by proteins. The participation of hnRNPs in each case may reflect the versatility of these proteins in linking sequence-specific nucleic acid binding with specific protein–protein interactions and serving as a fulcrum for multilateral molecular cross-talk (58). It seems highly unlikely that protein-mediated recoding will be restricted to the two present examples, given the plethora of hnRNPs and other RNA binding proteins present in host and viral proteomes.

## Supplementary Material

SUPPLEMENTARY DATA
