# Supplementary material for: A novel role for poly(C) binding proteins in programmed ribosomal frameshifting
Source: Nucleic Acids Res. 2016 Jun 2;44(12):5491–503. doi: 10.1093/nar/gkw480 (PMC4937337; doi:10.1093/nar/gkw480)
Supplement: SUPPLEMENTARY DATA [file supp_44_12_5491__index.html]

A novel role for poly(C) binding proteins in programmed ribosomal frameshifting — A novel role for poly(C) binding proteins in programmed ribosomal frameshifting — SUPPLEMENTARY DATA 

# A novel role for poly(C) binding proteins in programmed ribosomal frameshifting

## SUPPLEMENTARY DATA

- SUPPLEMENTARY DATA
