# Supplementary material for: A novel role for poly(C) binding proteins in programmed ribosomal frameshifting
Source: Nucleic Acids Res. 2016 Jun 2;44(12):5491–503. doi: 10.1093/nar/gkw480 (PMC4937337; doi:10.1093/nar/gkw480)
Supplement: SUPPLEMENTARY DATA [file supp_gkw480_nar-01168-h-2016-File009.pdf]

SUPPLEMENTARY INFORMATION

accompanying the main manuscript entitled

A novel role for poly(C) binding proteins in programmed ribosomal frameshifting.

Sawsan Napthine[1], Emmely E. Treffers[2], Susanne Bell[1], Ian Goodfellow[1], Ying Fang[3], Andrew E. Firth[1†], Eric J. Snijder[2†] and Ian Brierley[1†]

[1]Department of Pathology, University of Cambridge, Cambridge, U.K., [2]Leiden University Medical Center, Leiden, The Netherlands, [3]Kansas State University, Manhattan, U.S.A.

Contents:

Supplementary Figures S1-S6
Table S1
Supplementary Method
Supplementary References

SUPPLEMENTARY FIGURES AND LEGENDS

FIGURE S1: related to Figure 1. Nsp1β does not modulate PRF at established ribosomal frameshifting signals, nor stimulate programmed stop codon readthrough.

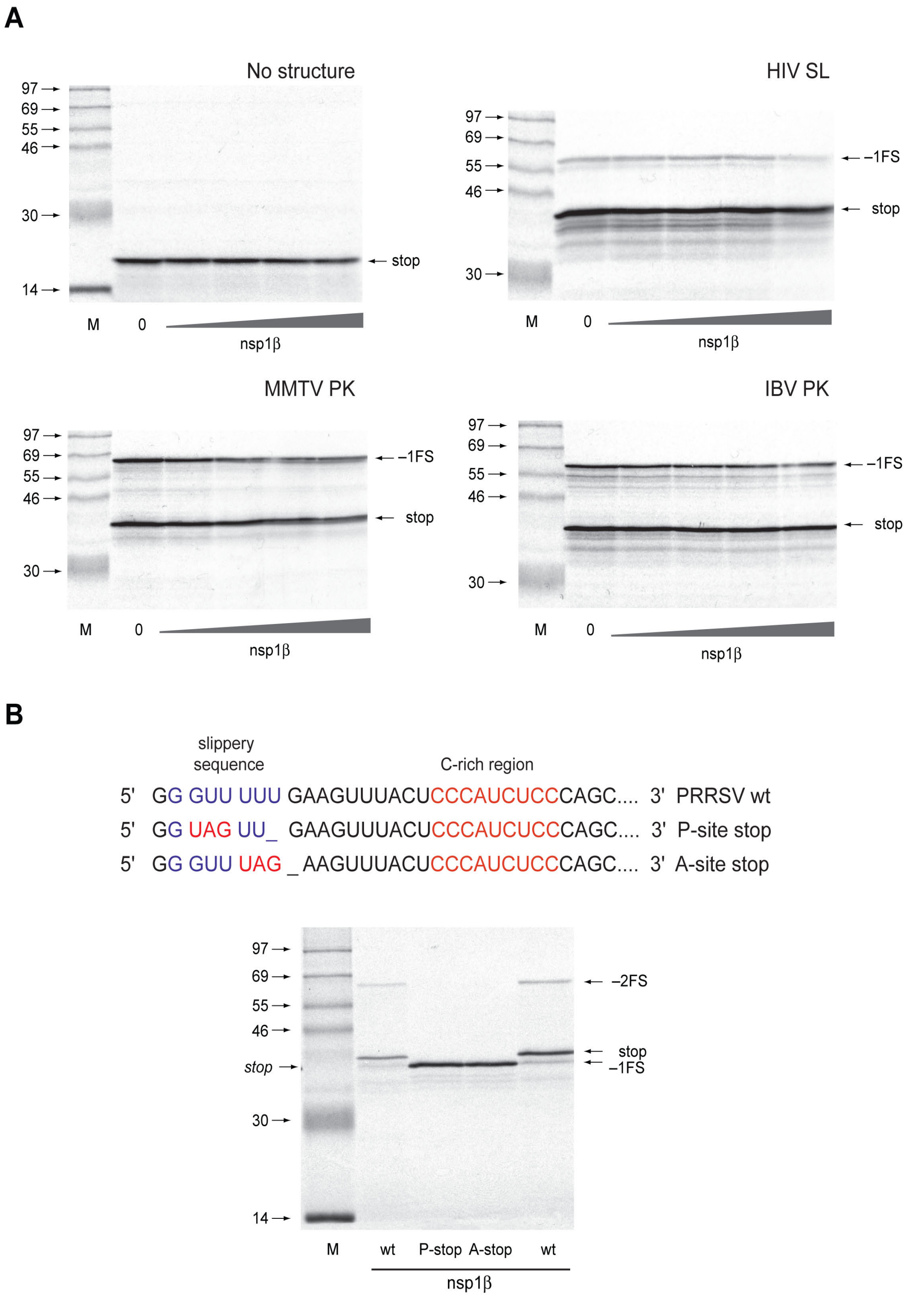

Figure S1 (A) Dual-luciferase frameshift reporter plasmids (Grentzmann et al., 1998; Fixsen et al. 2010) harbouring the –1 PRF signals of human immunodeficiency virus *gag/pol* (HIV; stem-loop stimulatory RNA; SL), mouse mammary tumor virus *gag/pro* (MMTV; pseudoknot stimulatory RNA; PK) (Girnary et al., 2007), infectious bronchitis virus *1a/1b* (IBV; PK) (Brierley et al., 1992) or the IBV slippery sequence (U_UUA_AAC) but lacking the PK (no structure) were transcribed *in vitro* and the resulting mRNAs translated in RRL in the absence or presence of nsp1β. The products were resolved by 15% SDS-PAGE and visualised by autoradiography. Molecular size markers were also run on the gel (M). Products derived from ribosomes that do not frameshift (stop) or that enter the −1 frame (–1 FS) are indicated. No specific inhibition or stimulation of frameshifting was seen, although some general inhibition of translation was

observed at the highest concentration of nsp1β.

(B) Variants of pDluc PRRSV/wt were prepared with an in-frame stop codon (UAG) placed within the slippery sequence at the position that would likely be decoded in the peptidyl- (P) or aminoacyl- (A) site of the ribosome during encounter of the nsp1β/PCBP complex. Transcribed mRNAs were translated in RRL in the presence of nsp1β alongside wild-type controls (wt). To align the stop codon with the outgoing –2 frame, a 3' base was deleted in each case (space underlined). In the configurations tested, no programmed readthrough event was detected.

Figure S2: related to Figure 3. Modulation of PRRSV –2/–1 PRF *in vivo* through siRNA-mediated knockdown of PCBPs.

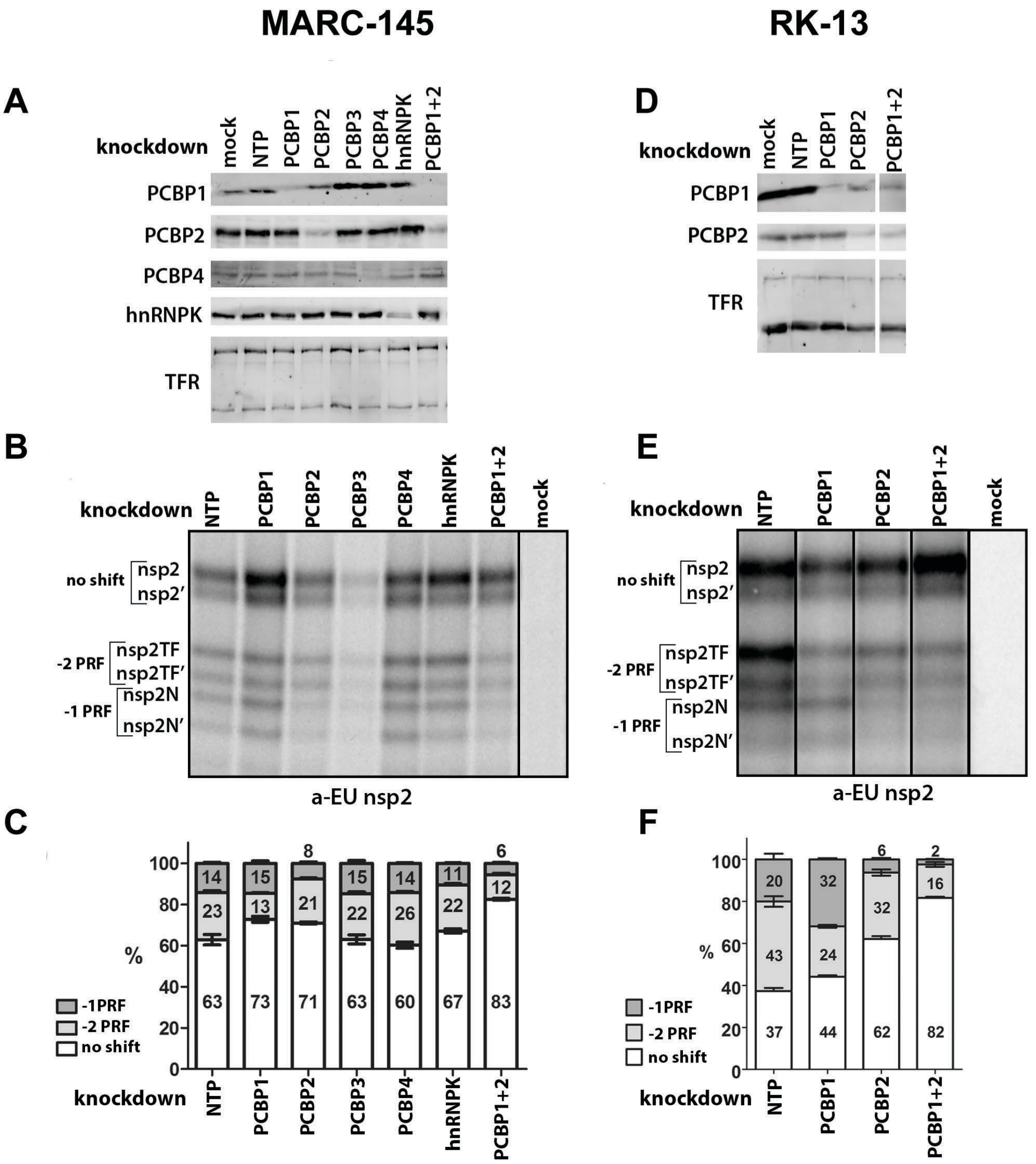

Figure S2 (A & D) MARC-145 (A) and RK-13 cells (D) were transfected with siRNA pools targeting PCBP1, PCBP2, PCBP3, PCBP4 or hnRNP K/J. Knockdown of proteins targeted by siRNAs was assessed by Western blotting (note: a suitable antibody for PCBP3 was not available). Knockdown of PCBP3, PCBP4 and hnRNPK resulted in an increase in PCBP1 expression, which might be a compensatory mechanism. In RK-13 cells, knockdown of PCBP2 also induced knockdown of PCBP1, possibly through an siRNA off-target effect (one of the siRNA sequences had only a single nt mismatch with rabbit PCBP1 mRNA and two mismatches with African green monkey PCBP1 mRNA).

(B & E) The PRF assay system used (Li et al., 2014) employs a recombinant vaccinia virus – T7 RNA polymerase

expression system to generate both the polyprotein fragment nsp1β-2 (which self-cleaves to provide proteins nsp1β and nsp2) and the encoding mRNA containing the PRRSV –2/–1 PRF signal. After metabolic labeling, expression products were immunoprecipitated with mAb α-EU-nsp2 that recognises the common N-terminal domain of nsp2, nsp2TF, and nsp2N. Immunoprecipitated proteins were separated by 6% SDS-PAGE and visualised by autoradiography to assess frameshifting efficiencies. Knockdown of PCBP3 in MARC-145 cells resulted in accelerated cell death during infection with recombinant vaccinia virus vT7-3, resulting in lower band intensities for the three nsp2 products upon immunoprecipitation compared to the other conditions.

(C & F) PRF efficiencies (%, y-axis) measured for each knockdown condition. Protein bands were quantified (triplicates) using ImageQuant TL software for MARC-145 cells (C) and RK-13 cells (F). The total intensity of all protein bands (nsp2, nsp2', nsp2TF, nsp2TF', nsp2N and nsp2N') was set as 100%. Frameshift efficiencies were calculated after normalisation for the methionine and cysteine content of each protein product. The products nsp2', nsp2TF' and nsp2N' are precursors. Note the baseline % FS differs in MARC-145 and RK-13 cells. Error bars represent SEM values (from triplicates).

Figure S3: related to Figure 4. Electrophoretic mobility shift analysis (EMSA) analysis of nsp1β/PCBP2 binding to the PRRSV –2/–1 PRF signal RNA and controls.

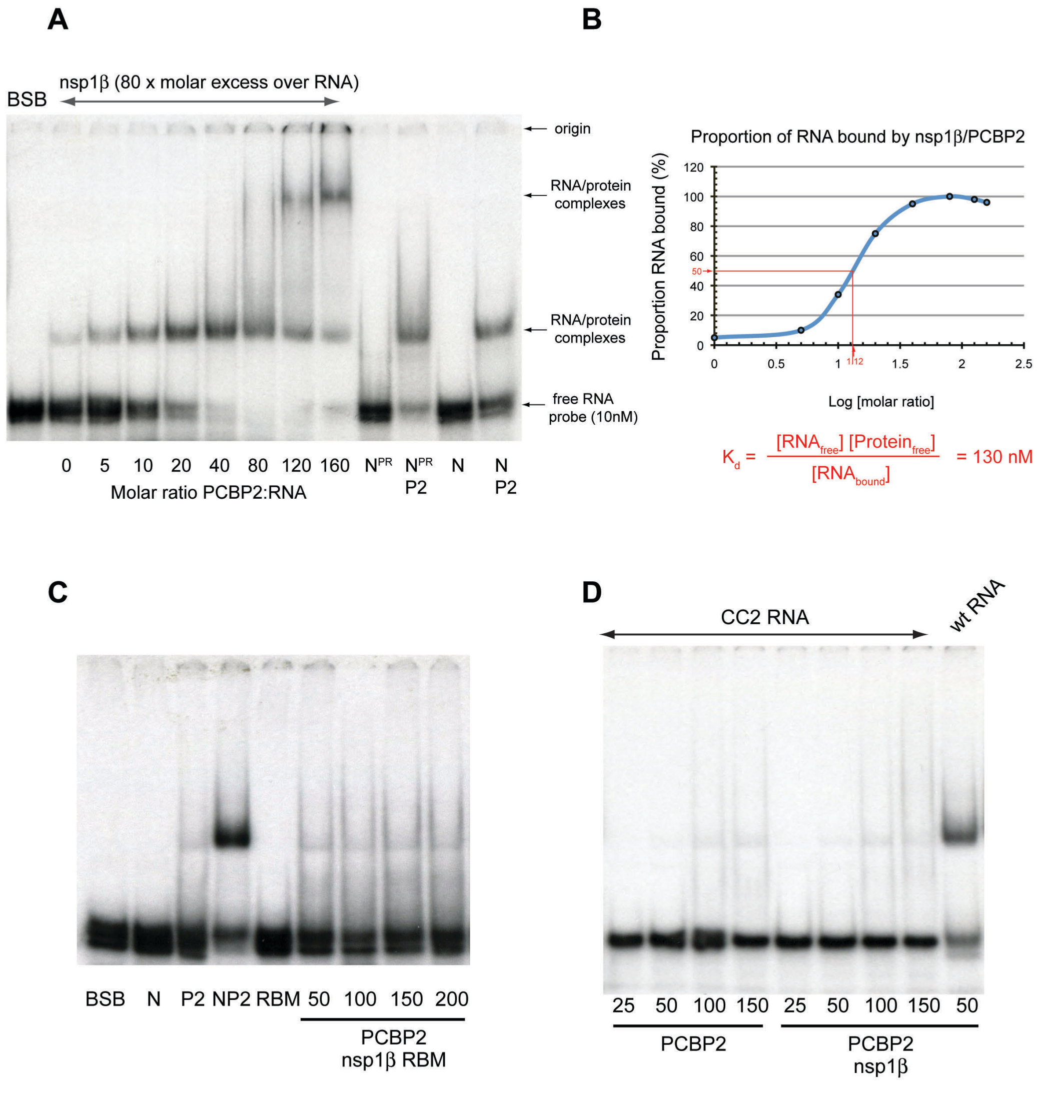

Figure S3 (A) *Left side.* EMSA titration of binding of nsp1β and PCBP2 to a short (58 nt) ^{32}P-labelled *in vitro* transcript containing the PRRSV PRF signal. After incubation at room temperature for 10 min, reactions were loaded onto a 4% non-denaturing polyacrylamide gel and following electrophoresis, the gel was fixed, dried and subjected to autoradiography. Nsp1β was present throughout at 0.8 µM and the numbers below lanes show fold molar excess of PCBP2 with respect to the radiolabelled RNA (10 nM). BSB: RNA was incubated alone with band-shift buffer (BSB).

Right side. Control EMSA showing similar RNA binding capacity of wild-type nsp1β/PCBP2 (NP2) and PR mutant nsp1β/PCBP2 ($N^{PR}P2$) complexes.

(B) RNA/protein complexes and free RNA were quantified by phosphorimagery and a binding curve plotted. From this, the Kd was estimated as 130 nM.

(C) Control EMSA reveals that nsp1β RBM mutant (at indicated molar excesses with respect to RNA) does not form stable RNA protein complexes in the absence (RBM, 1μM) or presence of PCBP2 (1 μM). Control lanes were nsp1β (N, 1 μM) and PCBP2 (P2, 1 μM) alone, both proteins together (NP2, 1 μM each) and BSB.

(D) Control EMSA confirms that RNA protein complexes do not form on the CC2 mRNA. Wild-type RNA (wt RNA) was used as a positive control.

Figure S4: related to Figure 4. Effect of competitor RNAs on –2/–1 PRF in PRRSV.

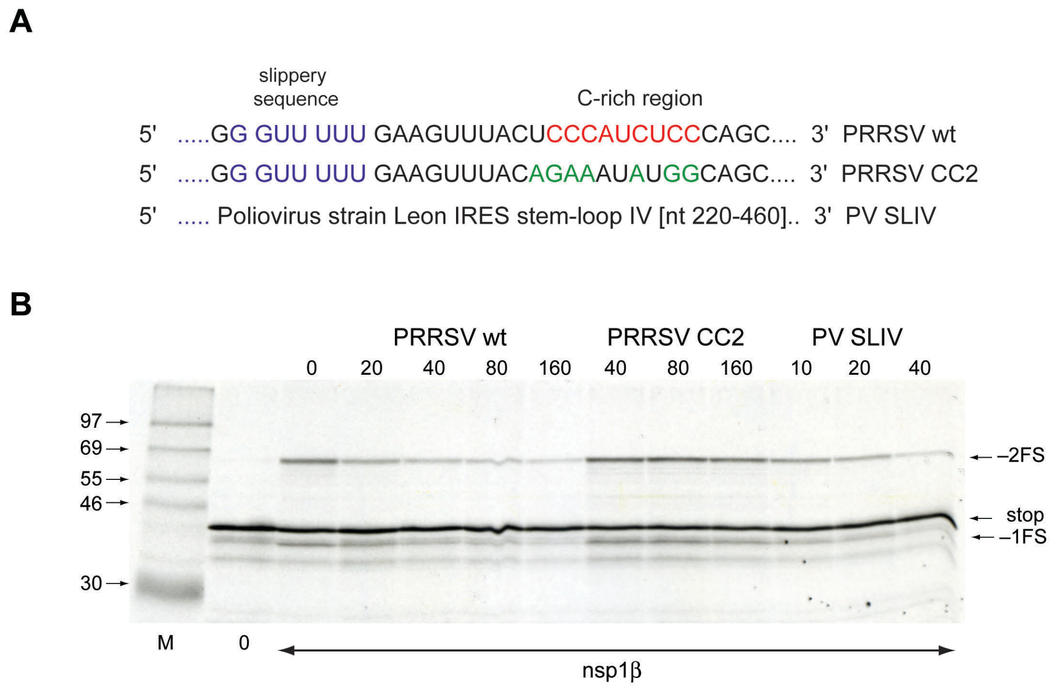

Figure S4 (A) Short, competitor RNAs containing the PRRSV PRF signal, the CC2 mutant or the poliovirus IRES stem-loop IV (P3/Leon/37; accession number K01392.1) were prepared by *in vitro* transcription.

(B) An mRNA (0.25 μM) derived from *FspI*-cut pDluc PRRSV/wt was translated in RRL in the presence of increasing molar excesses of competitor RNAs (numbered above the relevant lanes). The products were resolved by 12% SDS-PAGE and visualised by autoradiography. Molecular size markers were also run on the gel (M). Products derived from ribosomes that do not frameshift (stop) or that enter the −1 or −2 frames are indicated.

Figure S5: related to Figure 5. EMSA analysis of PCBP1 KH domain mutants.

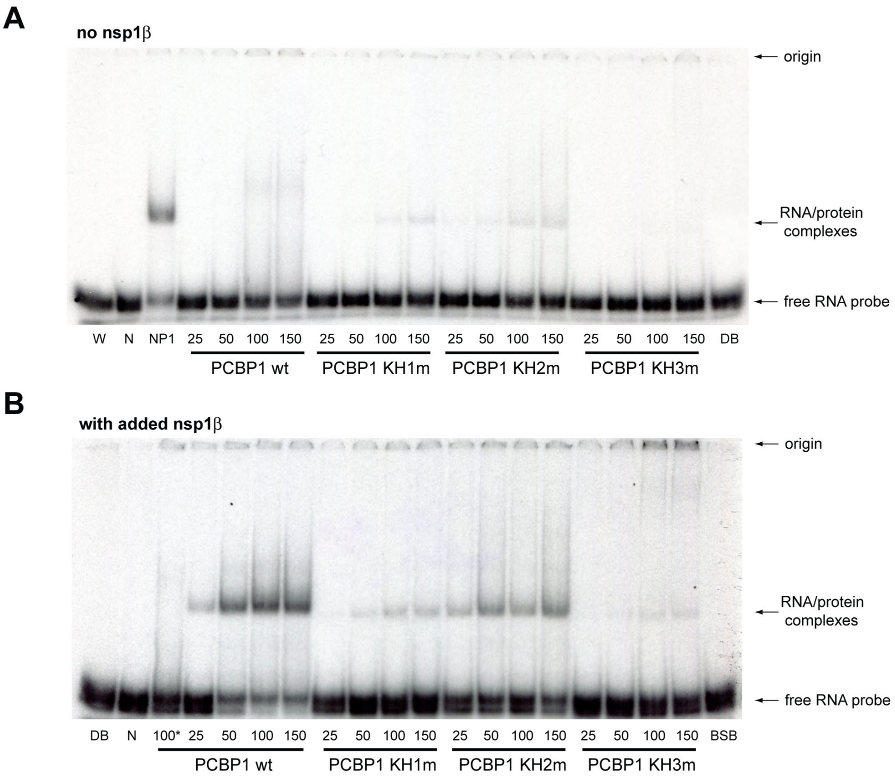

Figure S5 (A) and (B) Binding of the PCBP1 KH domain mutants to the PRRSV RNA (10 nM) was investigated in the absence (A) or presence (B) of added nsp1β (1 μM).

Figure S6: related to Figures 4 and 5.

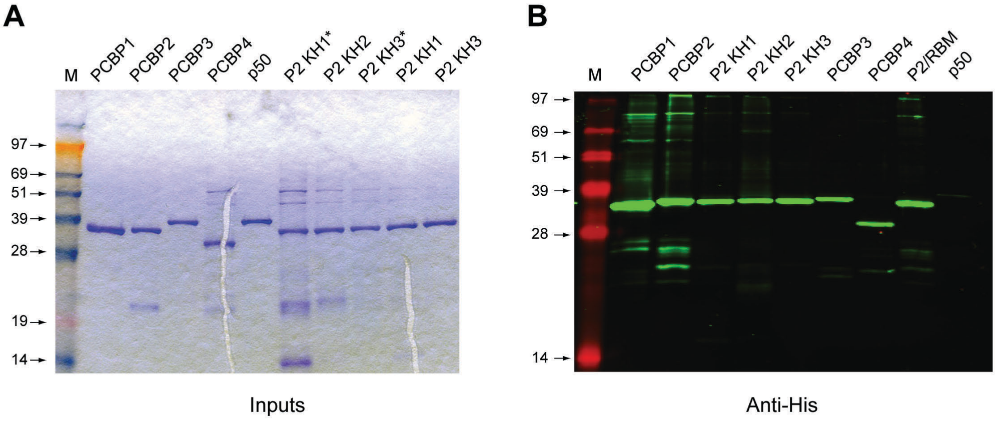

Figure S6 (A) GST-nsp1β WT was immobilised on glutathione-agarose beads and incubated individually with His-tagged PCBPs or a control protein (His-tagged central fragment of eIF4G, p50). Equivalent volumes of the incubated proteins were analysed by 12% SDS-PAGE and the gel stained with Coomasie blue (Inputs).

(B) Following a detergent wash, bound PCBPs were eluted and detected in Western blots with an anti-His antibody. In panel A, duplicate samples P2 KH1 and P2 KH3 (asterisked) were not used in the pull-downs. In panel B, P2/RBM represents the eluate from an incubation of PCBP2 with immobilised GST-nsp1β RBM. Note that the PCPB4 isoform employed (transcript variant X7; XM_006713271.1; encoding 346 amino acids) lacks the C-terminal 57 amino acids in

comparison to transcript variant 3 (Makeyev et al., 2002; encoding 403 amino acids), but retains the KH3 domain.

SUPPLEMENTARY TABLE

Frameshift efficiencies (triplicates ±SEM)

mRNA	Details	–2 PRF (%)	–1 PRF (%)
Figure 1			
PRRSV/wt	RRL + nsp1β	17.4±1.5	7.0±0.7
PRRSV/stop	RRL + nsp1β	15.8±1.3	7.8±0.7
PRRSV/wt	RRL + nsp1β PR	16.8±1.6	7.8±0.6
PRRSV/wt	RRL + nsp1β RBM	<2	<2
Figure 2			
PRRSV/U1	RRL + nsp1β	21.2±1.8	8.3±0.7
PRRSV/U2	RRL + nsp1β	19.6±2.0	7.9±0.5
PRRSV/G3	RRL + nsp1β	9.8±1.1	4.4±0.6
PRRSV/G4	RRL + nsp1β	<2	<2
PRRSV/G5	RRL + nsp1β	<2	<2
PRRSV/G6	RRL + nsp1β	4.6±0.8	3.8±0.5
PRRSV/G7	RRL + nsp1β	<2	<2
PRRSV/G8	RRL + nsp1β	4.8±0.6	3.5±0.4
PRRSV/G9	RRL + nsp1β	2.4±0.4	3.8±0.4
PRRSV/G10	RRL + nsp1β	11.2±1.2	4.3±0.2
PRRSV/G11	RRL + nsp1β	<2	<2
PRRSV/G12	RRL + nsp1β	<2	<2
PRRSV/G13	RRL + nsp1β	9.7±0.9	4.2±0.3
PRRSV/G14	RRL + nsp1β	19.2±1.7	5.8±0.5
Figure 3			
PRRSV/wt	RRL + nsp1β	25.0±2.2	8.1±0.7
PRRSV/wt	RRL + nsp1β + PCBP2	23.8±2.3	26.5±3.0
PRRSV/wt	WG + nsp1β	<2	<2
PRRSV/wt	WG + PCBP2	<2	<2
PRRSV/wt	WG + nsp1β + PCBP1	27.2±2.6	16.6±1.6
PRRSV/wt	WG + nsp1β + PCBP2	14.6±0.9	32.6±4.1
PRRSV/wt	WG + nsp1β + PCBP3	18.7±2.1	25.5±3.4
PRRSV/wt	WG + nsp1β + PCBP4	<2	<2
PRRSV/wt	WG + nsp1β + hnRNPK	<2	<2
Figure 5			
PRRSV/wt	WG + nsp1β + P1	26.9±1.9	23.0±2.1
PRRSV/wt	WG + nsp1β + $P1^{KH1}$	2.4±0.3	2.3±0.2
PRRSV/wt	WG + nsp1β + $P1^{KH2}$	23.5±3.3	18.8±1.9
PRRSV/wt	WG + nsp1β + $P1^{KH3}$	<2	<2
PRRSV/wt	WG + nsp1β + P2	14.9±1.5	39.0±3.3
PRRSV/wt	WG + nsp1β + P2	18.7±1.7	43.0±3.9
PRRSV/wt	WG + nsp1β + $P2^{KH1}$	6.4±0.6	31.3±2.8
PRRSV/wt	WG + nsp1β + $P2^{KH2}$	14.5±2.1	28.8±2.3
PRRSV/wt	WG + nsp1β + $P2^{KH3}$	3.1±1.1	<2
PRRSV/wt	WG + nsp1β + P1	17.2±1.6	20.2±1.5
PRRSV/wt	WG + nsp1β + $P1^{KH2}$	18.8±2.0	21.6±1.8
PRRSV/wt	WG + nsp1β + $P1^{KH1}$	<2	<2
PRRSV/wt	WG + nsp1β + $P1^{KH1}$ + $P1^{KH3}$	3.5±0.6	4.6±0.7
PRRSV/wt	WG + nsp1β + $P1^{KH3}$	<2	<2
PRRSV/wt	WG + nsp1β + P2	9.5±1.1	37.1±5.1
Figure 6			
Spacer 9nt	WG + PCBP1	26.9±2.4	3.6±0.4
Spacer 10nt	WG + PCBP1	34.5±2.4	20.1±3.2
Spacer 11nt	WG + PCBP1	48.8±5.1	3.1±0.1
Spacer 9nt	WG + PCBP2	17.8±2.4	12.5±0.7
Spacer 10nt	WG + PCBP2	14.3±1.9	20.0±2.0
Spacer 11nt	WG + PCBP2	2.1±0.1	14.8±1.4

SUPPLEMENTARY METHOD

siRNA-mediated knockdown

To determine frameshifting efficiencies after siRNA-mediated knockdown of PCBP expression, 6x10^4 MARC-145 or 4.8 x 10^4 RK-13 cells were seeded per well in 12-well clusters in DMEM containing 8% FCS. MARC-145 cells were transfected with siGENOME Human siRNA SMARTpools (Dharmacon) targeting PCBP1, PCBP2, PCBP3, PCBP4, or hnRNPK (final concentration 10 nM) using 2 µl of Dharmafect1 lipofection reagent (Dharmacon) per well. RK-13 cells were transfected with siRNA pools targeting PCBP1 or PCBP2 (final concentration 25 nM) using 3 µl Lipofectamine 2000 (LifeTechnologies) per well. A non-targeting pool (NTP) of "scrambled" siRNAs (Dharmacon) was used as a negative control. At 24 h post transfection (p.t.), the transfection medium was replaced with DMEM containing 8% FCS. Possible cytotoxic effects of siRNA transfection were monitored at 48 h p.t., using the CellTiter 96® AQueous Non-Radioactive Cell Proliferation Assay (Promega). After 120 min, the reaction was stopped by the addition of 25 µl of 10% SDS and absorbance at 490 nm (A490) was measured using a 96-well plate reader (Berthold). At 48 h p.t., the cells were infected with a T7 RNA polymerase-expressing recombinant vaccinia virus and 1 h later transfected with plasmid pL-nsp1β-2. Four hours later, the cells were starved for 30 min in methionine- and cysteine-free DMEM (Gibco) containing 2% FCS prior to a 45- (MARC-145) or 30-min (RK-13) metabolic labeling with 500 µCi/mL of a [^{35}S]Met/Cys mixture (EXPRE^{35}S^{35}S Protein Labeling Mix; Perkin-Elmer). Subsequently, cells were washed twice with PBS and harvested in lysis buffer (20 mM Tris, pH 7.6, 150 mM NaCl, 1% v/v NP-40, 0.1% DOC, 0.1% SDS and Complete protease inhibitor (Roche)).

The human sequences targeted by the siRNA pools were compared with the sequences of the African green monkey (*Chlorocebus sabaeus*) genes encoding PCBP1 (Genbank accession XM_007970341), PCBP2 (Genbank accession XM_008003436), PCBP3 (Genbank accession XM_007970531), PCBP4 (Genbank accession XM_007984435) and hnRNPK (Genbank accession XM_007969613) and the rabbit (*Oryctolagus cuniculus*) PCBP1 and PCBP2 genes (Genbank accession NM_001082124 and XM_002711018, respectively).

Following siRNA pool transfection, MARC-145 and RK-13 cells were harvested at 48 h p.t. or 72 h p.t./24 h p.i. by first washing with PBS and then lysing in 4x Laemmli's sample buffer (100 mM Tris-HCl, pH 6.8, 40% glycerol, 8% SDS, 40 mM DTT, 0.04 mg/ml bromophenol blue). Proteins were visualised by Western blot analysis as described previously (Treffers et al. 2015) using primary antibodies mAb-nsp2 Eu58-46, mAb-nsp1β Eu22-28 (Li et al., 2014), rabbit polyclonal anti-hnRNP E1 (C-terminal) (PCBP1) (Sigma-Aldrich), mouse monoclonal anti-PCBP2 (M07) (Abgent), rabbit polyclonal anti-human MGC10 (PCBP4) (PromoKine), mouse monoclonal anti-hnRNPK (Abcam), or mouse monoclonal antibody H68.4 against the transferrin receptor (Invitrogen) diluted in PBST containing 1% casein. Biotin-conjugated swine anti-rabbit (DAKO) or goat anti-mouse (DAKO), and Cy3-conjugated mouse anti-biotin (Jackson, Pennsylvania, USA) diluted in PBST containing 0.5% casein, were used for fluorescent detection with a Typhoon-9410 imager (GE Healthcare, UK).

Lysates from [^{35}S]Met/Cys-labelled cells were used to immunoprecipitate nsp2, nsp2TF and nsp2N using mAb-nsp2 Eu58-46 (Li et al. 2012) which recognizes the common N-terminal domain of the three products. Proteins were separated on a 6% SDS-PAGE gel and imaged as described previously (Li et al. 2014, as above) . Band intensities of nsp2, nsp2′, nsp2TF, nsp2TF′, nsp2N and nsp2N′ (nsp2′, nsp2TF′ and nsp2N′ are faster migrating forms of the three products; Fang et al, 2012) were quantified using ImageQuant TL (GE Healthcare) and normalised using the Met+Cys content of the respective products, while assuming that [^{35}S]Met and [^{35}S]Cys are incorporated with an efficiency ratio of 73:22 (the Met:Cys ratio in the mixture according to the manufacturer's documentation). Using these values, –2 PRF efficiencies were calculated as (nsp2TF + nsp2TF′)/(nsp2 + nsp2′ + nsp2TF + nsp2TF′ + nsp2N + nsp2N′) and –1 PRF efficiencies were calculated as (nsp2N + nsp2N′)/(nsp2 + nsp2′ + nsp2TF + nsp2TF′ + nsp2N + nsp2N′). Quantification was performed in triplicate.

SUPPLEMENTARY REFERENCES

Brierley,I., Jenner,A.J. and Inglis,S.C. (1992) Mutational analysis of the 'slippery-sequence' component of a coronavirus ribosomal frameshifting signal. *J. Mol. Biol.*, ***227***, 463-479.

Girnary,R., King,L., Robinson,L., Elston,R. and Brierley,I. (2007) Structure-function analysis of the ribosomal frameshifting signal of two human immunodeficiency virus type 1 isolates with increased resistance to viral protease inhibitors. *J. Gen. Virol.*, ***88***, 226-235.

Grentzmann,G., Ingram,J.A., Kelly,P.J., Gesteland,R.F. and Atkins,J.F. (1998) A dual-luciferase reporter system for studying recoding signals. *RNA*, ***4***, 479-486.

Treffers.E.E., Tas,A., Scholte,F.E., Van,M.N., Heemskerk,M.T., de Ru,A.H., Snijder,E.J. van Hemert,M.J. and van Veelen,P.A. (2015) Temporal SILAC-based quantitative proteomics identifies host factors involved in chikungunya virus replication. *Proteomics,* **15**, 2267-2280.
